# Sexual victimisation, peer victimisation, and mental health outcomes among adolescents in Burkina Faso: a prospective cohort study

**DOI:** 10.1016/S2215-0366(23)00399-1

**Published:** 2024-02

**Authors:** Kirsty S Lee, Dieter Wolke, Till Bärnighausen, Lucienne Ouermi, Mamadou Bountogo, Guy Harling

**Affiliations:** a Department of Psychology, University of Warwick, Warwick, UK; b Division of Health Sciences, Warwick Medical School, Warwick, UK; c Institute for Global Health, University College London, London, UK; d Africa Health Research Institute (AHRI), Durban, South Africa; e Heidelberg Institute of Global Health (HIGH), University of Heidelberg, Heidelberg, Germany; f Harvard TH Chan School of Public Health, Boston, MA, USA; g Département de Santé Publique, University of Ouagadougou, Ouagadougou, Burkina Faso; h Centre de Recherche en Santé de Nouna, Nouna, Burkina Faso; i School of Nursing and Public Health, University of KwaZulu-Natal, KwaZulu-Natal, South Africa; j MRC/Wits Rural Public Health and Health Transitions Research Unit, University of Witwatersrand, Johannesburg, South Africa

## Abstract

**Background:**

Sexual victimisation and peer victimisation are pervasive and increase risk for mental illness. Longitudinal studies that compare their unique and cumulative effects are scarce and have been done predominantly in high-income countries. The aims of this study were to examine the prevalence, prospective associations, and gender differences in sexual and peer victimisation and mental health in a low-income, African setting.

**Methods:**

In this prospective cohort study, data were obtained from the 2017 ARISE Adolescent Health Study, a population-representative, two-wave, prospective study of adolescents (aged 12–20 years) from Burkina Faso. A random sample of adolescents was drawn from ten villages, selected to capture the five main ethnic groups, and from one of the seven sectors of Nouna town, Burkina Faso, at two timepoints: Nov 12 to Dec 27, 2017, and Nov 15 to Dec 20, 2018. Standardised interviews were conducted in French or a local language by trained researchers. We measured victimisation exposure as sexual victimisation, peer victimisation, and polyvictimisation, using lifetime frequency of exposure, and we measured mental health symptoms and disorders using the Kutcher Adolescent Depression Scale, the Primary Care Post-Traumatic Stress Disorder screen IV and 5, and a question on lifetime self-harm and number of incidents in the past year. We calculated prevalence of victimisation and mental health symptoms and disorders at the two timepoints, and we used lifetime victimisation at the first timepoint to predict mental health at the second timepoint using logistic and negative binomial regressions. Gender differences were examined using interaction terms.

**Findings:**

Of 2544 eligible adolescents, 1644 participated at time 1 and 1291 participated at time 2. The final sample with data at both timepoints included 1160 adolescents aged 12–20 years (mean 15·1, SE 0·2), of whom 469 (40·4%) were girls and 691 (59·6%) were boys. The majority ethnic group was Dafin (626 [39·1%]), followed by Bwaba (327 [20·5%]), Mossi (289 [16·0%]), Samo (206 [13·0%]), Peulh (166 [9·7%]), and other (30 [1·6%]). After survey weight adjustment, sexual victimisation (weighted percentages, time 1, 256 [13·8%] of 1620; time 2, 93 [7·2%] of 1264) and peer victimisation (weighted percentages, time 1, 453 [29·9%] of 1620; time 2, 272 [21·9%] of 1264) were common, whereas polyvictimisation was more rare (weighted percentages, time 1, 116 [6·6%] of 1620; time 2, 76 [5·7%] of 1264). Longitudinally, sexual victimisation was associated with probable clinical disorder (adjusted odds ratio 2·59, 95% CI 1·15–5·84), depressive symptoms (adjusted incidence rate ratio [aIRR] 1·39, 95% CI 1·12–1·72), and symptoms of post-traumatic stress disorder (aIRR 2·34, 1·31–4·16). Peer victimisation was associated with symptoms of post-traumatic stress disorder (aIRR 1·89, 1·13–3·17) and polyvictimisation was associated with depressive symptoms (aIRR 1·34, 1·01–1·77). Girls reported more sexual victimisation (weighted percentages, 130 [17·3%] of 681 *vs* 126 [11·4%] of 939), boys reported more peer victimisation (weighted percentages, 290 [33·1%] of 939 *vs* 163 [25·2%] of 681), and there was a significant interaction between lifetime victimisation and gender for probable clinical disorder (F [degrees of freedom 7, sample 376] 2·16; p=0·030).

**Interpretation:**

Sexual and peer victimisation were common in the study setting and increased risk for mental health problems. Adolescent girls who have been sexually victimised are especially at risk of mental health problems. Interventions targeting sexual and peer violence in low-income settings are needed.

**Funding:**

Alexander von Humboldt Foundation, the Wellcome Trust, Fondation Botnar, and Harvard TH Chan School of Public Health.

## Introduction

Sexual violence is a worldwide public health problem; by age 18 years, 18% of girls and 8% of boys report sexual victimisation.^1^ Sexual violence can be perpetrated by family members, non-familial adults, intimate partners, peers, or strangers, and it can be psychological (eg, threats or harassment) or physical (eg, groping or forced penetration).^2^ Sexual victimisation has been associated with wide-ranging consequences, including risk for depression, post-traumatic stress disorder, and self-injurious behaviour.^3^, ^4^ The consequences of sexual victimisation might affect girls more than boys,^5^ but evidence is mixed.^6^

**Research in context**

**Evidence before this study**

Sexual victimisation and peer victimisation are global phenomena that affect a substantial proportion of young people and increase the risk for mental health problems. However, most research has been done in high-income settings, and there has been no direct comparison of the effects of sexual victimisation and peer victimisation on mental health outcomes in prospective studies. We did a systematic search in MEDLINE and PsycINFO to identify literature from inception to July 20, 2023, using the search string “(bulli* OR bully* OR peer victim*) AND (sexual harass* OR sexual violence OR sexual assault OR rape) AND (depress* OR ptsd OR self-harm OR self-injurious behaviour)”. We identified 56 peer-reviewed articles in MEDLINE and 87 peer-reviewed articles in PsycINFO, one of which prospectively compared the long-term effects of trauma (including peer and sexual victimisation) on psychotic symptoms in the UK. Victimisation was associated with psychotic symptoms, and effects were larger for repeated victimisation and polyvictimisation.

**Added value of this study**

To our knowledge, this is the first study to report the prevalence of sexual and peer victimisation and their unique and cumulative effects on mental health outcomes in Burkina Faso. Sexual and peer victimisation were prevalent in this low-income sample, and sexual victimisation was consistently associated with poor mental health and probable clinical disorder. Peer victimisation had few long-term effects on mental health in this sample, which might be a result of measurement, the outcomes evaluated, or local contextual factors. Adolescents, especially girls, who report sexual victimisation are at increased risk of adverse mental health outcomes.

**Implications of all the available evidence**

The current findings and previous research highlight the harmful effects of sexual victimisation. The prevalence estimates reported here will be valuable for estimating the demand needs for adolescent violence prevention in low-income settings. Approximately one in five girls and one in ten boys reported sexual victimisation, with the prevalence for boys in this sample being higher than worldwide estimates. Most research and public policy on sexual violence prevention is targeted towards girls and women, and although their need is greater, boys also require access to sexual assault services and should be included in future research. Peer victimisation was associated with mental health problems contemporaneously but had few long-term effects in this sample. Future research into bullying would therefore benefit from assessing other forms of violence concurrently and longitudinally, especially sexual victimisation, and reverse causality. Child and adolescent mental health legislation, strategy, and expenditure are scarce in Africa, and investment in violence prevention efforts could reduce child and adolescent mental health problems in low-income settings.

Another common form of violence is peer victimisation (ie, being bullied by people of a similar age), which affects about 30% of young people (aged <18 years) globally.^7^ Bullying is defined as intentional and repeated harm towards a peer^8^ and can be physical (eg, hitting and kicking), relational (eg, spreading a rumour), or verbal (eg, name calling). Being bullied increases the risk of adverse outcomes across social, educational, and health domains^9^ and causal effects have been shown on depression and self-harm.^10^

Despite sexual victimisation and peer victimisation being global phenomena, most research has been done in high-income settings, with a notable gap for low-income and middle-income countries (LMICs). Data on the epidemiology of victimisation in LMICs are scarce but suggest that peer victimisation in Africa is particularly high (46%), especially among boys.^11^ Polyvictimisation (defined as several forms of victimisation) in LMICs is high (38%) and a major contributor to mental illness.^12^ In Burkina Faso, psychological (22·7%) and physical violence (9·1%) are common and appear to be gender biased (eg, exposure to physical violence is higher among girls than boys).^13^

Because sexual and peer victimisation affect mental health, an examination of their unique and cumulative effects is warranted. It is unknown whether poor mental health is attributable uniquely to sexual victimisation, uniquely to peer victimisation, or accumulates from polyvictimisation. Attempts to disentangle the consequences of sexual and peer victimisation have been impeded by use of cross-sectional data,^4^ the absence of direct comparison using the same frequency timeframe,^14^ reliance on parent reports,^15^ or disregard of cumulative effects,^16^ and emphasis has been on high-income settings.^16^ Methodologically robust, prospective research in LMICs is thus needed to inform policy and prevention.

In a low-income, relatively rural setting in Burkina Faso, we investigated the prevalence of lifetime victimisation (sexual victimisation, peer victimisation, or polyvictimisation) and mental health outcomes (probable clinical disorder, symptoms of depression, post-traumatic stress disorder, and self-harm) across two annual surveys in a cohort of adolescents. We also investigated associations between lifetime victimisation and mental health outcomes 1 year later, and moderation by gender. We expected that sexual victimisation would be more prevalent among girls, that peer victimisation would be more prevalent among boys, and that there would be unique and cumulative effects on mental health.^4^, ^11^

## Methods

### Study design and participants

In this prospective cohort study, data were obtained from the Burkina Faso group of the ARISE Adolescent Health Study.^17^ For this subgroup, on Oct 25, 2017, a two-part stratified random sample of adolescents (aged 12–20 years) was drawn from the 2015 census of the Nouna Health and Demographic Surveillance System, overseen by the Centre de Recherche en Santé de Nouna (CRSN), which consisted of 59 villages and Nouna town, Burkina Faso. A random sample of 1795 adolescents was drawn from ten villages, selected to capture the five main ethnic groups. An additional 749 adolescents were randomly selected from one of the seven sectors of Nouna town. Follow-up was done 12 months later and 2544 adolescents were eligible to participate at time 1 (Nov 12 to Dec 27, 2017) or time 2 (Nov 15 to Dec 20, 2018). Standardised interviews were done privately in French or a local language by trained research assistants using tablet computers. Adolescents could decline to answer any question, and any urgent issues were passed to the CRSN team. As accurate measures of violence can be difficult to obtain (eg, because of stigmatisation or social undesirability), adolescents were randomly assigned to verbal response or non-verbal response^18^ for highly sensitive items (eg, sexual victimisation), and the effect of this response was analysed in a sensitivity analysis (appendix p 6). All procedures at time 1 and time 2 complied with the Declaration of Helsinki (1975, updated 2008). Study approvals were obtained from the CRSN Institutional Ethics Committee, village elders, participants (written consent or assent), parents or guardians if participants were younger than 18 years, and the Ethics Committee of the University of Heidelberg (time 2 only).

### Measures

Sexual victimisation was assessed using an escalating set of four experiences as follows: “someone made verbal jokes about wanting to have sex with you”; “someone touched you on your genitals or breast when you did not want to be touched”; “someone forced you to have sex against your will but you escaped”; “someone forced you to have sex against your will”. Responses were dichotomous (no or yes), and a response of yes to any item was recorded as exposure to lifetime sexual victimisation.

At time 1, adolescents were provided a definition of bullying as follows: “bullying occurs when a person or group of people say or do bad and unpleasant things to another person. It is also bullying when a person is teased a lot in an unpleasant way or when a person is left out of things on purpose. It is not bullying when two kids of about the same strength or power argue or fight or when teasing is done in a friendly and fun way”. They were then asked, “have you ever been bullied?” with an answer of yes or no, and “during the past 30 days, how many days were you bullied?”. Exposure to lifetime peer victimisation was recorded if adolescents responded yes to having ever been bullied. This response allowed for a direct comparison with sexual victimisation using the same marker of frequency (lifetime). At time 2, adolescents completed the Bullying and Friendship Interview,^19^ which contained three items on specific forms of bullying (direct, relational, or cyber), responded to on a 4-point scale from 0 (never) to 3 (at least once per week). For consistency with time 1, exposure to lifetime peer victimisation was recorded if adolescents reported any victimisation on any item.

At time 1 and time 2, adolescents were asked, “what was the main way you were bullied in the past 30 days?” with the answer options being physical, racial, religion, sexual, social exclusion, appearance, or other.

To examine unique and cumulative effects, sexual and peer victimisation were combined to create a categorical lifetime victimisation variable with four levels as follows: no victimisation; sexual victimisation only; peer victimisation only; and polyvictimisation (sexual and peer victimisation).

To assess mental health outcomes, a range of measures were used. The short version of the Kutcher Adolescent Depression Scale (KADS) was used to examine depression; this contains six items measuring symptoms over the past week.^20^ Responses were scored from 0 (hardly ever) to 3 (all of the time) and were summed to a total symptoms score. At time 1, adolescents were incorrectly given five response options (never, rarely, sometimes, most of the time, and always), so “never” and “rarely” were merged into “hardly ever” to restore comparability with the original scale. Internal consistency for the amended scale at time 1 was α=0·69 and the original scale at time 2 was α=0·73.

At time 1, the Primary Care Post-Traumatic Stress Disorder (PC-PTSD-IV) screen was used,^21^ which contains four items (nightmares, avoidance, hypervigilance, and numbness), and dichotomous (yes or no) responses were computed to a total symptoms score. At time 2, the PC-PTSD-5 was used, which included additional items on a triggering event and guilt. For consistency, we calculated the symptom scores for time 1 and time 2 using the four repeated items. Internal consistency for the original scale at time 1 was α=0·65 and the amended scale at time 2 was α=0·61.

Adolescents were given an explicit description of self-harm to ensure comprehension of the question “have you ever intentionally cut or burned your wrist, arms, or other area(s) of your body, or stuck sharp objects into your skin such as needles, pins, staples—not including tattoos, ear piercing, needles used for drugs, or body piercing or body modification—without any intent to die?”. Adolescents who responded yes were asked to report the number of self-harm incidents in the past 12 months, which was used as the outcome variable (no self-harm was coded as 0).

We identified the risk of probable clinical disorder using threshold cutoffs for disorder as follows: depression scores higher than 6 on the KADS;^19^ post-traumatic stress disorder scores of 3 or higher on the PC-PTSD-IV screen;^21^ and DSM-5 partial criteria of five incidents of self-harm in 1 year to indicate non-suicidal self-injury disorder. Adolescents who scored higher than any clinical cutoff were identified as having a probable clinical disorder, whereas symptoms of each disorder were defined as the secondary outcomes.

Data for the ARISE study^17^ were collected across many sites in Africa and the measures chosen were mostly validated somewhere in the continent but not typically in any one country. However, because Burkina Faso alone has about 70 languages, validation of the scales was not part of the protocol.

### Statistical analysis

Control variables included gender, age, fighting, and household wealth. Gender (boy or girl) and age were assessed by the interviewer and confirmed by the adolescent. As violence perpetration is associated with victimisation and mental health problems,^9^ we controlled for self-reported fighting (“during the past 12 months, how many times were you in a physical fight? A physical fight occurs when two people of about the same strength or power choose to fight each other”). Household wealth quintile was computed following procedures used in the Demographic and Health Surveys.^17^

Inverse sample weights accounting for differential participation by gender, age, religion, ethnicity, and village or town were calculated to adjust for survey non-response at time 1 and time 2. To avoid confounding, adolescents who responded that the main way they had been bullied was sexual were not included in the main analyses (appendix p 5).

Analyses were done using Stata version 18.0. Non-response patterns and descriptive data were examined using independent sample *t* tests and χ^2^ tests. First, we calculated prevalence (proportions) of lifetime victimisation and mental health at time 1 and time 2. Second, we examined longitudinal associations between victimisation at time 1 (sexual, peer, or polyvictimisation) and mental health at time 2, using logistic regression for the primary outcome, probable clinical disorder, and the secondary outcomes of depressive symptoms, post-traumatic stress disorder symptoms, and self-harm symptoms were analysed with negative binomial regressions given that they had an overdispersed count distribution. We ran unadjusted bivariate models, then multivariable models controlling for age, wealth, fighting, and time-1 mental health problems. Third, we examined gender differences by including a gender-by-victimisation interaction term in the unadjusted models.

Supplementary analyses (appendix pp 1–8) were done to examine: contemporaneous associations at time 1 and time 2; stricter cutoffs for victimisation, which were an exposure to sexual assault (items 2–4 on the sexual victimisation scale) or bullying by peers at least four times in the past 30 days (eg, about once per week); the inclusion of adolescents who were sexually bullied; the effect of verbal or non-verbal response randomisation; stratification by age, using a dummy variable of early (aged 12–14 years), mid (aged 15–17 years), and late (aged 18–20 years) adolescence; stratification by school attendance; and missing data.

### Role of the funding source

The funders of the study had no role in study design, data collection, data analysis, data interpretation, or writing of this report.

## Results

Of 2544 eligible adolescents, 1644 participated at time 1 and 1291 participated at time 2. Non-participation was primarily because the respondent could not be located (time 1, 58·5%) or had moved residence (time 1, 28·8%; time 2, 78·5%). Participation at time 2 was associated with gender, age, school status, ethnicity, and lifetime victimisation (table 1). The final sample of individuals at both timepoints who had data on predictors and outcomes included 1160 adolescents aged 12–20 years (mean 15·1, SE 0·2), of whom 469 (40·4%) were girls and 691 (59·6%) were boys. The majority ethnic group was Dafin with 626 (39·1%) of 1644 individuals, followed by Bwaba with 327 (20·5%), Mossi with 289 (16·0%), Samo with 206 (13·0%), Peulh with 166 (9·7%), and other ethnic groups with 30 (1·6%; all percentages are weighted).

**Table 1:** Dropout analyses at time 1 according to core variables and control variables between those who only completed time 1 versus those who also completed time 2

	**Statistical test results**		**p value**
	Test result	Degrees of freedom	
Gender	8·83*	1	0·0030
Age	4·93†	2541	<0·0001
Currently in school	34·15*	1	<0·0001
Physical fighting	−0·51†	1632	0·608
Religion	5·54*	3	0·136
Ethnicity	14·32*	5	0·014
Lifetime victimisation	10·27*	3	0·016
Probable clinical disorder	2·78*	1	0·096
Depression symptoms	0·73†	1629	0·465
Post-traumatic stress disorder symptoms	1·86†	1585	0·062
Self-harm symptoms	0·18†	1571	0·858

* χ^2^ test result.

† *t* test result.

After survey weight adjustment, at time 1 approximately half of the sample (825 [50·3%] of 1620) reported victimisation, 256 (13·8%) of 1620 reported sexual victimisation, 453 (29·9%) of 1620 reported peer victimisation, and 116 (6·6%) of 1620 reported polyvictimisation (table 2; all percentages are weighted). Prevalences of sexual victimisation, peer victimisation, and polyvictimisation at time 2 were lower than at time 1. At time 1, 113 (7·1%) of 1594 of the sample met criteria for probable clinical disorder versus 81 (5·1%) of 1247 at time 2 (table 2). Prevalence of probable depression was 1·3% at time 1 versus 1·2% at time 2, prevalence of probable post-traumatic stress disorder was 5·3% at time 1 versus 3·6% at time 2, and prevalence of partial criteria for non-suicidal self-injury disorder was 1·2% at time 1 versus 0·7% at time 2.

**Table 2:** Descriptive statistics of core variables and control variables, stratified by gender, for all available data

		**Total (N=1644)**	**Girls (N=696)**	**Boys (N=948)**
Age, years		1644;* 15·0 (0·2)	696; 15·0 (0·2)	948; 15·1 (0·2)
Education				
	Currently in school	822 (50·0%)	381 (54·7%)	441 (46·5%)
	Not in school	822 (50·0%)	315 (45·3%)	507 (53·5%)
Fighting		1643; 0·61 (0·05)	695; 0·41 (0·07)	948; 0·74 (0·07)
Religion				
	Muslim	1137 (68·3%)	478 (70·4%)	659 (66·9%)
	Catholic	340 (21·5%)	150 (20·7%)	190 (22·0%)
	Protestant	114 (6·7%)	45 (5·1%)	69 (7·8%)
	Animist	53 (3·5%)	23 (3·8%)	30 (3·3%)
Ethnicity				
	Bwaba	327 (20·5%)	143 (17·9%)	184 (22·3%)
	Dafin	626 (39·1%)	249 (37·7%)	377 (40·0%)
	Mossi	289 (16·0%)	145 (20·7%)	144 (12·7%)
	Peulh	166 (9·7%)	70 (11·1%)	96 (8·8%)
	Samo	206 (13·0%)	72 (10·2%)	134 (14·9%)
	Other	30 (1·6%)	17 (2·4%)	13 (1·2%)
Lifetime victimisation at time 1				
	No victimisation	795 (49·7%)	338 (51·4%)	457 (48·5%)
	Sexual victimisation only	256 (13·8%)	130 (17·3%)	126 (11·4%)
	Peer victimisation only	453 (29·9%)	163 (25·2%)	290 (33·1%)
	Polyvictimisation	116 (6·6%)	50 (6·2%)	66 (6·7%)
Mental health at time 1				
	Probable clinical disorder	1594 (7·1%)	677 (9·0%)	917 (5·8%)
	Depression	1631; 0·59 (0·04)	684; 0·64 (0·07)	947; 0·56 (0·06)
	Post-traumatic stress disorder	1623; 0·55 (0·03)	687; 0·71 (0·05)	936; 0·44 (0·04)
	Non-suicidal self-injury disorder	1607; 0·26 (0·03)	682; 0·29 (0·06)	925; 0·24 (0·03)
Lifetime victimisation at time 2				
	No victimisation	823 (65·3%)	332 (63·5%)	491 (66·4%)
	Sexual victimisation only	93 (7·2%)	66 (12·2%)	27 (3·8%)
	Peer victimisation only	272 (21·9%)	87 (17·9%)	185 (24·6%)
	Polyvictimisation	76 (5·7%)	36 (6·4%)	40 (5·2%)
Mental health at time 2				
	Probable clinical disorder	1247 (5·1%)	510 (5·8%)	737 (4·6%)
	Depression	1288; 1·02 (0·04)	529; 1·14 (0·07)	759; 0·94 (0·05)
	Post-traumatic stress disorder	1268; 0·20 (0·02)	521; 0·22 (0·04)	747; 0·19 (0·03)
	Non-suicidal self-injury disorder	1270; 0·23 (0·03)	518; 0·28 (0·06)	752; 0·19 (0·03)

Data are presented as n; mean (SE) for continuous data or n (%). All results are adjusted for survey weights, and so percentages might differ from those expected with the denominator.

* Overall mean age is different here for all data compared with overall age for the core sample cited in the text.

Lifetime victimisation was associated with mental health outcomes in the unadjusted (table 3) and adjusted (table 4) models, with effects marginally attenuated in the adjusted models. In the adjusted models, sexual victimisation was associated with probable clinical disorder, depressive symptoms, and symptoms of post-traumatic stress disorder. Peer victimisation was only associated with post-traumatic stress disorder, and polyvictimisation was only associated with depressive symptoms (table 4). There were no prospective associations between victimisation and self-harm.

**Table 3:** Unadjusted effects of lifetime victimisation on mental health 1 year after victimisation was reported for the total sample, stratified by gender

	**Total (N=1160)**	**Girls (n=469)**	**Boys (n=691)**
**Probable clinical disorder, OR (95% CI)**			
Sexual victimisation only	3·44 (1·67–7·09)	4·18 (1·46–11·93)	2·86 (0·99–8·24)
Peer victimisation only	1·78 (0·92–3·50)	2·11 (0·70–6·38)	1·63 (0·71–3·76)
Polyvictimisation	2·62 (1·00–6·94)	5·35 (1·46–19·58)	1·28 (0·27–6·17)
**Depressive symptoms, IRR (95% CI)**			
Sexual victimisation only	1·46 (1·17–1·82)	1·73 (1·29–2·33)	1·13 (0·85–1·52)
Peer victimisation only	1·00 (0·83–1·20)	1·04 (0·75–1·46)	0·98 (0·78–1·24)
Polyvictimisation	1·40 (1·01–1·84)	1·33 (0·86–2·07)	1·45 (1·02–2·06)
**Symptoms of post-traumatic stress disorder, IRR (95% CI)**			
Sexual victimisation only	2·76 (1·60–4·75)	2·51 (1·10–5·74)	3·02 (1·46–6·24)
Peer victimisation only	1·72 (1·06–2·81)	1·91 (0·95–3·85)	1·63 (0·84–3·15)
Polyvictimisation	0·55 (0·62–3·92)	3·90 (1·46–10·39)	0·16 (0·02–1·13)
**Self-harm symptoms, IRR (95% CI)**			
Sexual victimisation only	1·65 (0·84–3·23)	2·43 (0·98–6·04)	0·88 (0·40–1·90)
Peer victimisation only	1·44 (0·83–2·52)	1·95 (0·69–5·50)	1·18 (0·68–2·07)
Polyvictimisation	1·12 (0·49–2·57)	1·89 (0·58–6·13)	0·66 (0·22–1·99)

Binary logistic regression and negative binomial regression models, corrected for survey non-response. No victimisation was used as the reference category. OR=odds ratio. IRR=incidence rate ratio.

**Table 4:** Adjusted effects of lifetime victimisation on mental health 1 year after victimisation was reported for the total sample, stratified by gender

	**Total (N=1160)**	**Girls (n=469)**	**Boys (n=691)**
**Probable clinical disorder, aOR (95% CI)**			
Sexual victimisation only	2·59 (1·15–5·84)	3·10 (0·92–10·52)	2·12 (0·63–7·09)
Peer victimisation only	1·75 (0·88–3·52)	1·12 (0·61–6·05)	1·66 (0·69–3·95)
Polyvictimisation	2·07 (0·72–5·97)	2·74 (0·67–16·81)	1·11 (0·23–5·43)
**Depressive symptoms, aIRR (95% CI)**			
Sexual victimisation only	1·39 (1·12–1·72)	1·51 (1·13–2·01)	1·16 (0·86–1·58)
Peer victimisation only	0·99 (0·82–1·19)	1·00 (0·72–1·38)	1·00 (0·79–1·27)
Polyvictimisation	1·34 (1·01–1·77)	1·20 (0·75–1·91)	1·48 (1·04–2·10)
**Symptoms of post-traumatic stress disorder, aIRR (95% CI)**			
Sexual victimisation only	2·34 (1·31–4·16)	2·29 (1·00–5·28)	2·64 (1·17–5·93)
Peer victimisation only	1·89 (1·13–3·17)	2·19 (1·13–4·26)	1·83 (0·86–3·90)
Polyvictimisation	1·10 (0·41–2·93)	2·82 (0·92–8·61)	0·14 (0·17–1·05)
**Self-harm symptoms, aIRR (95% CI)**			
Sexual victimisation only	1·60 (0·79–3·25)	2·26 (0·83–6·14)	0·80 (0·36–1·79)
Peer victimisation only	1·37 (0·77–2·45)	1·97 (0·73–5·33)	1·07 (0·61–1·89)
Polyvictimisation	1·07 (0·47–2·43)	1·79 (0·51–6·20)	0·66 (0·21–2·05)

Negative binomial regression models corrected for survey non-response. Adjusted models controlled for age, wealth, fighting, and mental health at time 1. No victimisation was used as the reference category in all analyses. Probable clinical disorder refers to any of the three outcomes using a clinical cutoff. Bivariate models and the covariate coefficients of adjusted models are shown in the appendix (pp 1–8). aOR=adjusted odds ratio. aIRR=adjusted incidence rate ratio.

Significant gender differences in victimisation prevalence at time 1 and time 2 were observed (χ^2^ [degree of freedom (df) 3] 14·98; p=0·002; table 2). At both timepoints, sexual victimisation was reported by more girls than boys, whereas peer victimisation was reported by more boys than girls. Prevalence of probable clinical disorder (χ^2^ [df 1] 5·24; p=0·02) and symptoms of post-traumatic stress disorder (t [df 1621] 5·86; p<0·001) at time 1 and depressive symptoms at time 2 (t [df 1286] 2·46; p=0·01) were higher among girls than boys (table 2).

Significant interactions between lifetime victimisation and gender were observed for probable clinical disorder (F [df 7, 376] 2·16; p=0·03), depressive symptoms (F [df 7, 376] 3·43; p=0·001), and symptoms of post-traumatic stress disorder (F [df 7, 376] 3·47; p=0·001). Girls were more likely to report victimisation and to experience more mental health problems than boys (figure). There was no significant interaction between victimisation and gender for self-harm (F [df 7, 376] 1·12, p=0·35).

**Figure:**
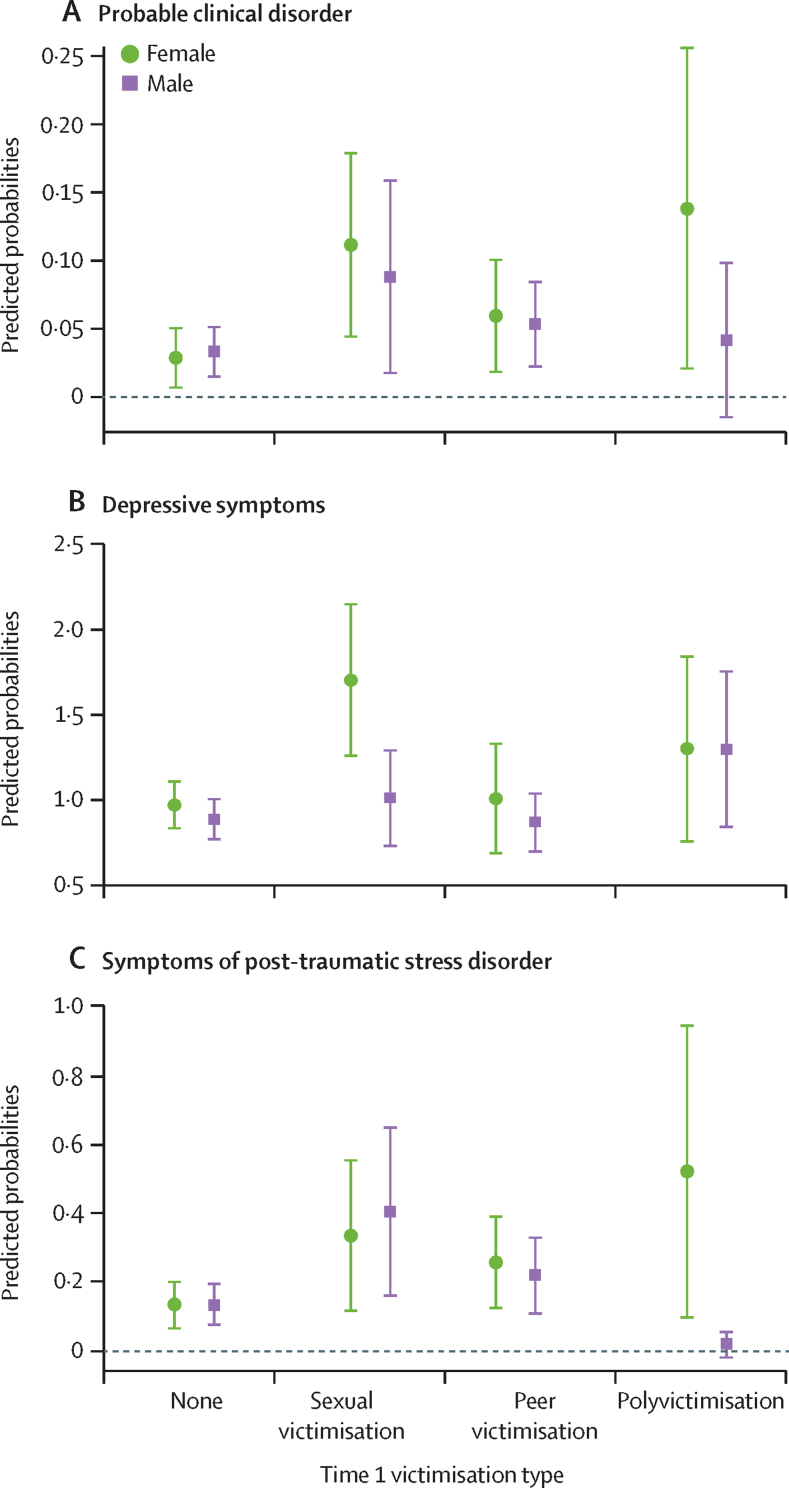
Interaction effects between gender and lifetime sexual victimisation, peer victimisation, and polyvictimisation on probable clinical disorder, depressive symptoms, and symptoms of post-traumatic stress disorder Error bars represent 95% CIs.

Supplementary analyses (appendix pp 1–8) did not change the primary results, but some differences were found: cross-sectional analyses showed stronger associations between peer victimisation and polyvictimisation and mental health outcomes; using strict cutoffs, symptoms of post-traumatic stress disorder were 3·8 times higher among boys reporting peer victimisation at least once per week; sexual victimisation and sexual bullying had a dose-response effect on depressive symptoms; non-verbal responses increased prevalence of sexual victimisation; early adolescents (aged 12–14 years) who reported polyvictimisation or sexual victimisation had a five times higher risk for probable clinical disorder and post-traumatic stress disorder, respectively; there were associations between sexual victimisation and mental health among children in school, and associations between peer victimisation and mental health among children not in school; and reports of victimisation and mental health within participants were largely consistent over time.

## Discussion

In a community of adolescents in Burkina Faso, approximately half of the sample reported lifetime victimisation at time 1 and a third of the sample at time 2. Girls and victimised youth were most likely to drop out (ie, highly vulnerable adolescents), which might explain lower prevalences of exposures and primary outcome at time 2. Sexual victimisation and peer victimisation were common, but fewer adolescents reported polyvictimisation. There were significant longitudinal associations between victimisation and mental health outcomes, with effects most consistently emerging for sexual victimisation, which was associated with probable clinical disorder, depressive symptoms, and symptoms of post-traumatic stress disorder. There were fewer significant longitudinal associations between peer victimisation and polyvictimisation and mental health outcomes, but there were consistent cross-sectional associations (appendix pp 2–3). Victimisation was not longitudinally associated with self-harm; however, self-harm was associated with peer victimisation contemporaneously at time 1 and time 2. Girls reported a higher prevalence of sexual victimisation, poorer mental health, and were more adversely affected following victimisation than boys.

Lifetime sexual victimisation in this sample was similar to worldwide prevalences,^1^ whereas peer-victimisation prevalence was lower than that previously reported in Burkina Faso,^13^ and aligned with international averages. Sexual and peer violence in LMICs are typically high^11^, ^22^ and tend to be higher in urban areas, which could partially explain this discrepancy in prevalence, given the relatively rural nature of our sample (ie, small villages and the isolated town of Nouna). Prevalence of probable clinical disorder (7·1% at time 1 and 5·1% at time 2) was marginally lower than the 9·5% prevalence reported in a meta-analysis of adolescents in sub-Saharan Africa using clinical diagnostic instruments.^23^ This difference might reflect our assessment of common problems, such as depression and post-traumatic stress disorder, but not other psychopathologies prevalent in Africa, such as anxiety or externalising problems.^23^, ^24^ Nonetheless, our findings show a considerable burden of victimisation experiences and mental health problems in this population.

A key aim of our study was to compare the associations between sexual and peer victimisation and mental health outcomes using the same frequency (lifetime). Sexual victimisation consistently predicted adverse mental health outcomes, such that the risk of probable clinical disorder was 2·6 times higher among adolescents who were sexually victimised, which is in line with the prevalence reported in previous work^3^ and highlights the need for sexual violence prevention. By contrast, there were few longitudinal associations between peer victimisation and mental health, nor cumulative effects from polyvictimisation. This was surprising given the substantial evidence of short-term and long-term adverse effects of peer victimisation,^9^ and dose-response associations between polyvictimisation and mental health.^12^ Possible explanations include low school attendance, or exposure to other stressors, such as patriarchal norms, poverty, death threats, or insurgency.^12^ Peer victimisation was associated with mental health outcomes cross-sectionally, especially self-harm, which could indicate that adverse consequences dissipate over time,^25^ or that adolescents experiencing mental health problems are targeted (reverse causality).^26^ Notably, mental health problems associated with peer victimisation were higher among children not in school, which could suggest important contextual differences in peer victimisation between high-income and low-income countries. Despite there being no statistical significance in the longitudinal analyses, effect sizes were often substantial, and if our results are replicated in similar samples, bullying interventions in LMICs could have meaningful benefits.

As expected, there were gender differences in prevalence and outcomes,^1^ which replicated findings in high-income contexts.^4^, ^27^ Sexual victimisation was more prevalent among girls than boys, but one in nine boys (11·4%) reported sexual victimisation, which is higher than the worldwide average.^1^ As in other LMICs, peer victimisation was more prevalent among boys than girls.^11^ Girls reported poorer mental health than boys, especially following victimisation, evidenced by significant interactions on probable clinical disorder, depressive symptoms, and symptoms of post-traumatic stress disorder. Most research and public policy on sexual violence prevention is targeted towards girls and women, but boys also require access to and inclusion in sexual assault services and research.

Despite the merits of this study, it is not without limitations. First, regarding instrumentation, we were unable to validate the scales in the target population, because Burkina Faso has about 70 languages. The time 1 bullying measure probably did not have sensitivity and specificity, and the absence of definitional consistency means that the term bullying itself might not have translated into the local context with fidelity. Sexual bullying typically refers to sexual name calling or rumour spreading (eg, real or perceived sexual behaviour or orientation), but we cannot be certain that this behaviour was being referenced in this LMIC context. Bullying measures that include behavioural descriptions, such as the measure we used at time 2,^21^ are recommended in future studies. The change in instrumentation of post-traumatic stress disorder between timepoints might have led to lower prevalence at time 2 and might have biased the estimates.^28^ Second, we broadly assessed peer and sexual victimisation to compare their effects accurately using the same frequency exposure (ever), but stricter cutoffs are typically used for bullying,^19^ and we found that peer victimisation every week resulted in a strong association with symptoms of post-traumatic stress disorder among boys. We excluded sexual bullying to avoid confounding and found that sexual victimisation and sexual bullying had a dose-response effect on depressive symptoms. However, this item only pertained to exposure over the past 30 days, so we cannot assume that instances before this window were captured. Future studies of victimisation should include measures using the same frequency markers. Third, we did not examine perpetration. Whether perpetrators of sexual violence are family members, partners, peers, or strangers has important implications for the strategic allocation of prevention resources. In terms of bullying, about 5% of adolescents report co-occurring victimisation and perpetration, and this group demonstrates the poorest outcomes across domains.^9^, ^14^ As a proxy, we included physical fighting, but this variable had no associations with the outcomes and the results were almost identical when we removed this variable from the analysis. Last, as is common in longitudinal studies, there was non-random attrition. Despite using inverse sampling weights, our effect sizes are probably attenuated. Because the analyses were exploratory, replication is encouraged.

Notwithstanding its limitations, this study has several clinical and policy implications. There are considerable sexual and peer victimisation and mental health problems in this population, and thus there is a clear need for locally developed policies for violence prevention. Child and adolescent mental health legislation, strategy, and expenditure are limited in Burkina Faso,^29^ yet our findings highlight the potential benefits of investing in mental health and violence-prevention services. As victimisation and mental health problems were a risk for children currently in and out of school, especially early (aged 12–14 years) adolescents and mid (15–17 years) adolescents, multicomponent, cross-context interventions that begin in childhood would be beneficial.

To our knowledge, this is the first study to identify representative rates of adolescent sexual and peer victimisation in Burkina Faso and is the first longitudinal study of unique and cumulative associations with mental health outcomes in an LMIC. Sexual and peer victimisation are highly prevalent in this deprived setting, and sexual victimisation is associated with a range of mental health problems, especially among girls. Although vulnerability to sexual and peer victimisation differed by gender, all adolescents need protection from violence. Our study provides novel and important findings for future policy and service planning in rural Africa and offers new insights for youth violence prevention researchers worldwide.

## Data sharing

Data are available on reasonable request. Data are not publicly available because consent was not given by participants for data to be shared openly. This is in part because entire age cohorts of some villages are included in the dataset, potentially allowing for deductive disclosure with sufficient local information. For this reason, anonymised data are available from the ARISE study data controllers only following signature of a data-use agreement restricting onward transmission. Anyone wishing to replicate the analyses presented or wishing to conduct further collaborative analyses using the ARISE study data (which are welcomed and considered on the basis of a letter of intent), should contact GH (g.harling@ucl.ac.uk) in the first instance.

## Declaration of interests

We declare no competing interests.
