# Supplementary material for: Sexual victimisation, peer victimisation, and mental health outcomes among adolescents in Burkina Faso: a prospective cohort study
Source: Lancet Psychiatry. 2024 Feb;11(2):134–42. doi: 10.1016/S2215-0366(23)00399-1 (PMC11932973; doi:10.1016/S2215-0366(23)00399-1)
Supplement: Supplementary appendix [file mmc1.pdf]

THE LANCET
Psychiatry

Supplementary appendix

This appendix formed part of the original submission and has been peer reviewed. We post it as supplied by the authors.

Supplement to: Lee KS, Wolke D, Bärnighausen T, Ouermi L, Bountogo M, Harling G. Sexual victimisation, peer victimisation, and mental health outcomes among adolescents in Burkina Faso: a prospective cohort study. *Lancet Psychiatry* 2024; **11:** 134–42.

Supplementary Online Content

Lee et al. Lifetime Sexual Victimisation, Peer Victimisation, and Polyvictimisation on Mental Health Outcomes Among Adolescents in Burkina Faso: A Prospective Cohort Study

eTable 1. Adjusted within-time associations between lifetime victimisation and mental health at T1.

eTable 2. Adjusted within-time associations between lifetime victimisation and mental health at T2.

eTable 3. Adjusted effects of severe victimisation on mental health one year later for the total sample and stratified by sex.

eTable 4. Adjusted effects of lifetime victimisation on mental health one year later for the total sample, including those who were sexually bullied by peers.

eTable 5. Adjusted lifetime victimisation on mental health one year later for the total sample, controlling for the effects of the nonverbal response card randomisation.

eTable 6. Adjusted effects of lifetime victimisation on mental health one year later, stratified by age.

eTable 7. Adjusted effects of lifetime victimisation on mental health one year later, stratified by schooling.

eTable 8. Adjusted residuals of the association between reports of victimisation at T1 and T2 for the complete case sample

eTable 1. Adjusted within-time associations between lifetime victimisation and mental health at T1.

	Probable clinical disorder aOR (95% CI)	Depressive symptoms aIRR (95% CI)	PTSD symptoms aIRR (95% CI)	Self-harm symptoms aIRR (95% CI)
Sexual victimisation only	2·27 (1·14-4·52)	1·21 (0·76-1·93)	1·81 (1·33-2·46)	1·49 (0·77-2·88)
Peer victimisation only	1·41 (0·79-2·53)	1·65 (1·23-2·22)	1·73 (1·36-2·19)	1·89 (1·17-3·06)
Polyvictimisation	2·89 (1·36-6·12)	1·82 (1·13-2·95)	2·06 (1·43-2·95)	2·27 (0·98-5·28)
Age	1·12 (1·00-1·24)	1·05 (0·98-1·12)	1·07 (1·02-1·12)	1·00 (0·89-1·12)
Wealth	1·04 (0·87-1·24)	1·02 (0·91-1·13)	1·14 (1·06-1·22)	1·03 (0·88-1·20)
Fighting	1·04 (0·93-1·17)	1·07 (0·97-1·18)	1·02 (0·95-1·08)	1·10 (0·97-1·24)

Note. Binary logistic regression and negative binomial regression models corrected for survey non-response. aOR: adjusted odds ratio; aIRR: adjusted incidence rate ratio; CI: confidence intervals. No victimisation was used as the reference category.

eTable 2. Adjusted within-time associations between lifetime victimisation and mental health at T2

	**Probable clinical disorder aOR (95% CI)**	**Depressive symptoms aIRR (95% CI)**	**PTSD symptoms aIRR (95% CI)**	**Self-harm symptoms aIRR (95% CI)**
Sexual victimisation only	6·40 (2·78-14·74)	1·23 (0·89-1·69)	3·58 (1·76-7·29)	3·77 (1·52-9·40)
Peer victimisation only	2·89 (1·50-5·59)	0·92 (0·76-1·12)	2·53 (1·67-3·81)	1·70 (1·05-2·74)
Polyvictimisation	6·24 (2·83-13·74)	1·45 (1·02-2·06)	6·19 (3·77-10·18)	1·86 (1·01-3·44)
Age	1·13 (1·00-1·27)	1·04 (1·00-1·08)	1·11 (1·00-1·23)	1·01 (0·91-1·12)
Wealth	1·11 (0·89-1·38)	0·98 (0·92-1·04)	1·15 (0·97-1·36)	1·07 (0·91-1·26)
Fighting	1·13 (0·99-1·28)	1·00 (0·95-1·05)	1·05 (0·92-1·21)	1·09 (0·94-1·26)

Note. Binary logistic regression and negative binomial regression models corrected for survey non-response. aOR: adjusted odds ratio; aIRR: adjusted incidence rate ratio; CI: confidence intervals. No victimisation was used as the reference category.

eTable 3. Adjusted effects of severe victimisation on mental health one year later for the total sample and stratified by sex.

	Total (N = 1160)	Girls (n = 469)	Boys (n = 691)
**Probable clinical disorder aOR (95% CI)**			
Sexual victimisation only	1·83 (0·96-3·48)	2·65 (1·00-7·02)	1·10 (0·42-2·91)
Peer victimisation only	1·25 (0·30-5·19)	3·20 (0·29-35·38)	0·76 (0·14-4·01)
Polyvictimisation	-	-	-
**Depressive symptoms aIRR (95% CI)**			
Sexual victimisation only	0·96 (0·81-1·14)	1·62 (0·99-2·63)	1·23 (0·92-1·63)
Peer victimisation only	1·11 (0·89-1·37)	1·24 (0·63-2·44)	0·98 (0·40-2·39)
Polyvictimisation	0·95 (0·61-1·47)	-	1·04 (0·73-1·48)
**PTSD symptoms aIRR (95% CI)**			
Sexual victimisation only	1·44 (0·86-2·41)	1·81 (0·87-3·74)	1·17 (0·66-2·08)
Peer victimisation only	2·04 (1·20-3·48)	2·35 (0·57-23·32)	3·82 (1·07-13·56)
Polyvictimisation	-	-	-
**Self-harm symptoms aIRR (95% CI)**			
Sexual victimisation only	1·27 (0·50-3·19)	1·57 (0·66-3·73)	0·73 (0·28-1·94)
Peer victimisation only	1·27 (0·58-2·74)	2·55 (0·95-6·84)	0·83 (0·15-4·75)
Polyvictimisation	1·30 (0·10-16·70)	-	2·46 (0·28-21·33)

Note. Binary logistic regression and negative binomial regression models corrected for survey non-response. aOR: adjusted odds ratio; aIRR: adjusted incidence rate ratio; CI: confidence intervals. No victimisation was used as the reference category. Adjusted models controlled for age, wealth, fighting and T1 mental health. Severe sexual victimisation was characterised as a positive response to any of the assault questions (items 2-4). Severe peer victimisation was characterised as a positive response to bullying by peers at least four times in the past 30 days (equivalent to once a week). Of the total sample at T1, severe sexual violence was reported by 19.2%, severe peer victimisation was reported by 2.2%, and severe polyvictimisation was reported by 0.5%. The low prevalence of polyvictimisation meant some estimates ‘could not be calculated.

eTable 4. Adjusted effects of lifetime victimisation on mental health one year later for the total sample, including those who were sexually bullied by peers.

	**Probable clinical disorder aOR (95% CI)**	**Depressive symptoms aIRR (95% CI)**	**PTSD symptoms aIRR (95% CI)**	**Self-harm symptoms aIRR (95% CI)**
Sexual victimisation only	2·47 (1·08-5·65)	0·97 (0·82-1·15)	2·28 (1·27-4·08)	1·56 (0·76-3·20)
Peer victimisation only	1·57 (0·79-3·15)	0·90 (0·80-1·01)	1·71 (1·02-2·86)	1·25 (0·70-2·23)
Polyvictimisation	2·08 (0·76-5·69)	0·93 (0·75-2·16)	1·14 (0·45-2·85)	1·04 (0·48-2·25)

Note. Binary logistic regression and negative binomial regression models corrected for survey non-response. aOR: adjusted odds ratio; aIRR: adjusted incidence rate ratio; CI: confidence intervals. No victimisation was used as the reference category. Adjusted models controlled for age, wealth, fighting and T1 mental health. At T1 n=35 (2.1%) adolescents reported that the main way in which they were bullied was sexual.

eTable 5. Adjusted lifetime victimisation on mental health one year later for the total sample, controlling for the effects of the nonverbal response card randomisation.

	**Probable clinical disorder aOR (95% CI)**	**Depressive symptoms aIRR (95% CI)**	**PTSD symptoms aIRR (95% CI)**	**Self-harm symptoms aIRR (95% CI)**
Sexual victimisation only	3·49 (1·69-7·21)	1·46 (1·17-1·82)	2·76 (1·60-4·76)	1·68 (0·86-3·27)
Peer victimisation only	1·81 (0·92-3·53)	1·00 (0·83-1·21)	1·74 (1·07-2·85)	1·46 (0·84-2·53)
Polyvictimisation	2·58 (0·99-6·76)	1·40 (1·06-1·83)	1·49 (0·59-3·74)	1·09 (0·48-2·46)
Nonverbal response card randomisation	0·69 (0·40-1·19)	0·96 (0·82-1·11)	0·87 (0·58-1·31)	0·82 (0·53-1·29)

Note. Binary logistic regression and negative binomial regression models corrected for survey non-response. aOR: adjusted odds ratio; aIRR: adjusted incidence rate ratio; CI: confidence intervals. No victimisation was used as the reference category.

eTable 6. Adjusted effects of lifetime victimisation on mental health one year later, stratified by age

	Early adolescents (12-14-years)	Mid adolescents (15-17-years)	Late adolescents (18-20-years)
	n = 741	n = 564	n = 339
**Probable clinical disorder aOR (95% CI)**			
Sexual victimisation only	2·82 (0·55-14·45)	3·86 (1·58-9·42)	1·11 (0·28-4·42)
Peer victimisation only	1·54 (0·50-4·73)	2·53 (1·05-6·12)	0·54 (0·06-4·89)
Polyvictimisation	5·62 (1·47-21·54)	0·77 (1·00-6·29)	1·31 (0·23-7·57)
**Depressive symptoms aIRR (95% CI)**			
Sexual victimisation only	1·19 (0·83-1·71)	1·62 (1·17-2·24)	1·32 (0·89-1·96)
Peer victimisation only	0·76 (0·59-0·98)	1·45 (1·01-2·07)	1·26 (0·86-1·83)
Polyvictimisation	1·30 (0·88-1·91)	1·74 (1·08-2·78)	1·09 (0·58-2·04)
**PTSD symptoms aIRR (95% CI)**			
Sexual victimisation only	5·21 (1·76-15·36)	2·71 (1·25-5·89)	1·12 (0·37-3·43)
Peer victimisation only	2·23 (0·84-5·94)	2·07 (1·00-4·30)	1·01 (0·33-3·09)
Polyvictimisation	2·04 (0·44-9·43)	0·42 (0·12-1·47)	2·92 (0·48-17·66)
**Self-harm symptoms aIRR (95% CI)**			
Sexual victimisation only	0·69 (0·15-3·07)	2·36 (0·92-6·01)	0·88 (0·29-2·62)
Peer victimisation only	1·54 (0·66-3·60)	1·59 (0·73-5·49)	0·84 (0·34-2·06)
Polyvictimisation	1·65 (0·37-7·44)	1·14 (0·38-6·44)	0·41 (0·12-1·39)

Note. Negative binomial regression models corrected for survey non-response. aIRR: adjusted incidence rate ratio; CI: confidence intervals. Adjusted models controlled for sex, wealth, fighting and T1 mental health. No victimisation was used as the reference category in all analyses. Probable clinical disorder refers to any of the three outcomes using a clinical cut-off.

eTable 7. Adjusted effects of lifetime victimisation on mental health one year later, stratified by schooling.

	Not in school	Currently in school
	n = 822	n = 822
**Probable clinical disorder aOR (95% CI)**		
Sexual victimisation only	2·02 (0·54-7·60)	2·79 (1·19-6·52)
Peer victimisation only	3·08 (1·12-8·45)	1·19 (0·48-2·96)
Polyvictimisation	2·81 (0·50-15·92)	1·90 (0·55-6·49)
**Depressive symptoms aIRR (95% CI)**		
Sexual victimisation only	1·43 (1·06-1·93)	1·35 (1·02-1·79)
Peer victimisation only	1·33 (0·99-1·77)	0·78 (0·62-0·98)
Polyvictimisation	1·71 (1·11-2·62)	1·06 (0·70-1·59)
**PTSD symptoms aIRR (95% CI)**		
Sexual victimisation only	1·54 (0·60-3·93)	3·16 (1·59-6·27)
Peer victimisation only	2·81 (1·35-5·84)	1·43 (0·70-2·92)
Polyvictimisation	1·94 (0·53-7·09)	0·50 (0·14-1·74)
**Self-harm symptoms aIRR (95% CI)**		
Sexual victimisation only	0·94 (0·37-2·39)	2·21 (0·91-5·38)
Peer victimisation only	1·29 (0·66-2·50)	1·59 (0·74-3·42)
Polyvictimisation	1·08 (0·41-2·86)	1·09 (0·25-4·71)

eTable 8. Adjusted residuals of the association between reports of victimisation at T1 and T2 for the complete case sample

		T2			
		No victimisation	Sexual victimisation	Peer victimisation	Poly-victimisation
T1	No victimisation	3·213	-3·038	0·240	-1·746
	Sexual victimisation	-3·577	3·428	-0·570	2·155
	Peer victimisation	-0·110	-0·035	0·899	-0·575
	Polyvictimisation	0·992	-0·878	-0·313	-0·271
